# Multi-omic Directed Networks Describe Features of Gene Regulation in Aged Brains and Expand the Set of Genes Driving Cognitive Decline

**DOI:** 10.3389/fgene.2018.00294

**Published:** 2018-08-09

**Authors:** Shinya Tasaki, Chris Gaiteri, Sara Mostafavi, Lei Yu, Yanling Wang, Philip L. De Jager, David A. Bennett

**Affiliations:** ^1^Rush Alzheimer’s Disease Center, Rush University Medical Center, Chicago, IL, United States; ^2^Department of Statistics, Department of Medical Genetics, University of British Columbia, Vancouver, BC, Canada; ^3^Center for Translational and Computational Neuroimmunology, Department of Neurology, Columbia University Medical Center, New York, NY, United States; ^4^Cell Circuits Program, Broad Institute, Cambridge, MA, United States

**Keywords:** Alzheimer’s dementia, cognitive decline, multi-omics data integration, gene regulatory network, xQTL, expression quantitative trait DNA methylation, expression quantitative trait histone acetylation, GWAS

## Abstract

Multiple aspects of molecular regulation, including genetics, epigenetics, and mRNA collectively influence the development of age-related neurologic diseases. Therefore, with the ultimate goal of understanding molecular systems associated with cognitive decline, we infer directed interactions among regulatory elements in the local regulatory vicinity of individual genes based on brain multi-omics data from 413 individuals. These local regulatory networks (LRNs) capture the influences of genetics and epigenetics on gene expression in older adults. LRNs were confirmed through correspondence to known transcription biophysics. To relate LRNs to age-related neurologic diseases, we then incorporate common neuropathologies and measures of cognitive decline into this framework. This step identifies a specific set of largely neuronal genes, such as *STAU1* and *SEMA3F*, predicted to control cognitive decline in older adults. These predictions are validated in separate cohorts by comparison to genetic associations for general cognition. LRNs are shared through www.molecular.network on the Rush Alzheimer’s Disease Center Resource Sharing Hub (www.radc.rush.edu).

## Introduction

The repeated failure of traditional drug discoveries for Alzheimer’s dementia (AD) (Cummings et al., 2014; Gauthier et al., 2016) indicate the necessity of a paradigm shift toward precision medicine that aims to perturb the right targets in specific people at the right time (Collins and Varmus, 2015). To pursue this goal, big biomedical data including genomes, epigenomes, transcriptomes, and proteomes have been generated by community aging studies and consortium efforts (Hodes and Buckholtz, 2016). In theory, the integration of multi-omics data provides the basis for a more complete and accurate understanding of complex molecular regulation and thus increases the odds of identifying effective therapeutic targets for patients with cognitive decline. However, in practice, the elucidation of integrated molecular regulatory mechanisms remains rare, especially in the context of the aging human brain. Generating such integrated mechanisms requires phenotypes and multiple omics assayed in the same set of individuals, the mathematical and biological frameworks to integrate these data, and external validation of results.

To provide integrated multi-omic molecular networks that are relevant to the aging brain, it is necessary to first quantify and validate cross-omics interactions from multiple omics gathered in the same set of individuals from longitudinal studies of aging. Then, utilizing these cross-omics interactions such as relationships of DNA methylation and histone acetylation to gene expression, we can accurately determine the relationships of genes to AD-related neuropathologies and cognitive decline. The caveat to cross-omic interactions from correlation-based analyses is that they are not necessarily causal relationships. Approaches which combine genetics with gene expression address this issue to provide directed gene interaction networks (Chaibub Neto et al., 2010; Zhang et al., 2013), while related approaches extend causality from genetics to disease phenotypes (Schadt et al., 2005).

We further develop an analytical framework for combining genetic and multiple types of omics data to infer mechanisms regulating gene expression levels in aged brains. This approach infers a series of biophysically based links from genetic variants, through multiple molecular traits to dementia-related phenotypes, by modeling local regulatory networks (LRNs), in the vicinity of individual genes. We extensively validate the output of these models in terms of known biological relationships between regulatory elements. Leveraging the inferred structure of the LRNs, we find the epigenetic modifications predicted to affect gene expression levels and show strong enhancer/repressor activities of those modifications by assessing overlaps with a variety of genomic annotations. Moreover, LRNs predict genes upstream of dementia-related phenotypes and we validate their genetic associations with general cognition in separate cohorts. Overall, this approach begins to address the multiple data integration challenges and the multi-layer regulation around genes with predicted associations to ongoing disease phenotypes. The results can increase the efficiency of experimental work by directing it toward upstream regulators that are likely to control cognitive decline and neuropathology in older individuals.

## Materials and Methods

### Cohort Summary

We infer LRN’s in the context of two longitudinal, community-based aging studies: the Religious Orders Study (ROS) and the Rush Memory and Aging Project (MAP), collectively referred to as ROSMAP (Bennett et al., 2018). Together, these ongoing studies have enrolled ∼3500 older persons without dementia, all of whom have agreed to brain donation and annual detailed clinical evaluation, cognitive testing and blood donation. The cognitive levels, cognitive decline, and pathological indices utilized in the LRNs all come directly from measurements provided by this cohort. All phenotypes and omics data are shared freely through the RADC hub www.radc.rush.edu.

### Standard Protocol Approvals, Registrations, and Patient Consents

The parent cohort studies and substudies were approved by Rush University Medical Center Institutional Review Boards. Participants provided written informed consent and all participants signed an Anatomic Gift Act for brain donation.

### Tau and β-amyloid Measurement

To quantify the burden of parenchymal deposition of β-amyloid and the density of abnormally phosphorylated paired helical filament tau (PHFtau)-positive neurofibrillary tangles, tissue was dissected from midfrontal cortex. 20 μm sections were stained with antibodies to the β-amyloid protein and the tau protein, and quantified with image analysis and stereology, as previously described (Bennett et al., 2006, 2012b; Schneider et al., 2012; Boyle et al., 2013). Briefly, β-amyloid was labeled with an antibody for β-amyloid (10D5; Elan, Dublin, Ireland; 1:1,000). Immunohistochemistry was performed using diaminobenzidine as the reporter, with 2.5% nickel sulfate to enhance immunoreaction product contrast. Between 20 and 90 video images of stained sections were sampled and processed to determine the average percent area positive for β-amyloid. PHFtau-tangles were labeled with an antibody specific for phosphorylated tau (AT8; Innogenetics, San Ramon, CA, United States; 1:1,000). Between 120 and 700 grid interactions were sampled and processed, using the stereological mapping station, to determine the average density (per mm^2^) of PHFtau-tangles.

### Cognitive Function Assessment

For each participant, comprehensive cognitive assessments were administered at baseline and during each annual follow-up visit. Details on cognitive assessment have been described previously (Wilson et al., 2002, 2003, 2015; Bennett et al., 2006, 2012a). Briefly, the battery contains a total of 17 cognitive performance tests which assess 5 dissociable cognitive domains including, episodic memory (7 measures), semantic memory (3 measures), working memory (3 measures), perceptual speed (2 measures), and visuospatial ability (2 measures). To minimize the floor and ceiling effects, composite measures were used to examine the longitudinal cognitive decline. For each test, raw scores were standardized using the baseline mean and standard deviation across the cohorts. The *z*-scores were subsequently averaged across all the 17 tests to obtain a summary measure representing global cognition. Similarly, summary measures for individual cognitive domains were obtained by averaging *z* scores from the corresponding tests. The longitudinal rate of decline was computed for each participant using linear mixed models, which estimate the mean rate of change for the sample as a whole, but allow positive or negative deviations for each individual and are less sensitive to the number of follow-up visits or missing data.

### Genotype Processing

Genotyping of the ROS and MAP subjects was performed on the Affymetrix Genome-Wide HumanSNP Array6.0 (*n* = 1709) and the Illumina OmniQuad Express platform (*n* = 382). DNA was extracted from whole blood, lymphocytes, or frozen brain tissue, as previously described (De Jager et al., 2012). To minimize population admixture, only self-declared non-Hispanic Caucasians were genotyped. At the sample level, samples with genotyping success rate <95%, discordant genetically inferred and reported gender, or excess inter/intra-heterozygosity were excluded. At the probe level, genotyping data from both platforms were processed with the same quality-control (QC) metrics: Hardy-Weinberg equilibrium *p* < 0.001, genotype call rate <0.95, misshap test <1 × 10^-9^. QC was performed using version 1.08p of the PLINK software. EIGENSTRAT was used with the default setting to remove population outliers. The resultant datasets include 729,463 single nucleotide polymorphisms (SNPs) for 1,709 individuals (Affy) and 624,668 SNPs for 382 individuals (Omni). Dosages for all SNPs on the 1000 Genomes reference were imputed using version 3.3.2 version of the BEAGLE software [1000 Genomes Project Consortium interim phase I haplotypes, 2011 Phase 1b data freeze(verify) data freeze]. The coordinate of SNPs was updated with dbSNP Build 150. SNPs with minor allele frequency greater than 0.05 and info score greater than 0.3 were used for the analysis, resulting in 7,159,943 SNPs.

### RNAseq Processing

Details on RNAseq are published (Ng et al., 2017; Mostafavi et al., 2018). Briefly, RNA from 540 individuals was extracted from the dorsolateral prefrontal cortex (DLPFC) with the miRNeasy mini kit (Qiagen, Venlo, Netherlands) and the RNase free DNase Set (Qiagen, Vento, Netherlands). RNA concentration was quantified using Nanodrop (Thermo Fisher Scientific, Waltham, MA, United States), and RNA quality was assessed using an Agilent Bioanalyzer. RNAseq was performed using Illumina HiSeq with 101 bp paired-end reads with an average depth of 90 m reads. The trimmed reads were aligned to the reference genome using Bowtie and the expression fragments per kilobase million (FPKM) values were estimated using RSEM. Samples from 508 individuals which have genotype data and pass the expression outlier test are further normalized. Only highly expressed genes were kept (mean expression >2 FPKM), resulting in 13,484 expressed genes for analysis. The FPKM values were log transformed and biological covariates and technical covariates were removed from gene expression data via linear regression. Biological covariates include sex, age at death, and three genotyping principal components (PCs). Technical covariates include post mortem interval (PMI), RNA integrity number (RIN), study index (ROS or MAP), and lab processing batch. In this study, the genomic coordinates coding genes were updated to Ensembl release 90 with the annotables R package for 13,412 genes. RNA-seq data of 413 individuals with both epigenome and SNP measurements undergo the cross-omic analysis.

### Methylation Processing

Details on DNA methylation data are published (De Jager et al., 2014). DNA from 740 individuals was extracted from DLPFC using the Qiagen QIAamp DNA mini protocol. DNA methylation data were generated using Illumina Infinium HumanMethylation450k Bead Chip assay. Raw data were further processed using Methylation Module v1.8 from the Illumina Genome Studio software suite to generate a beta value for each cytosine guanine dinucleotide. The Illumina 450K platform contains a mixture of “type 1” and “type 2” probes which have distinct methylation levels that can negatively affect the analysis, so we used the wateRmelon R-package to account for this mixture and process all raw 450K arrays into Beta methylation values. Next, we performed an initial data reduction using the minfi R package to collapse adjacent probes with similar methylation levels into single units. This reduced the ∼450K methylation probes to 194,244 DNA 5C methylation (DNAm) clusters, which we refer to simply as DNAm loci. Samples from 663 individuals which have genotype data were used for further normalization step. Then, Beta methylation values were converted to *M*-values (Du et al., 2010) and quantile normalized. To remove outlier samples based on DNA methylation data, the statistic di was calculated and samples with a di value outside of 1.5x the interquartile range (*n* = 27) were excluded. Then, biological covariates and technical covariates were removed from DNA methylation data via linear regression. Biological covariates include sex, age at death, cell epigenotype specific indexes, and three genotyping PCs. Technical covariates include PMI, variables related to the position of arrays, study index (ROS or MAP), and lab processing batch. DNA methylation data of 413 individuals with other omics measurements are used in the cross-omic analysis.

### Histone Acetylation Processing

Details on histone acetylation data are published (Ng et al., 2017; Klein et al., 2018; Mostafavi et al., 2018). Gray matter was dissected on ice from 714 biopsies of DLPFC. The tissue was minced and crosslinked with 1% formaldehyde at room temperature and then homogenized in a cell lysis buffer. Then the nuclei were lysed in nuclei lysis buffer and chromatin was sheared by sonication. Chromatin was incubated overnight with the anti-H3K9Ac mAb (Millipore, Bedford, MA, United States) and purified with protein A sepharose beads. The final DNA was extracted and used for Illumina library construction following usual methods of end repair, adapter ligation and gel size selection. Samples were pooled and sequenced with 44 bp single end reads on the Illumina HiSeq. Single-end reads were aligned by the BWA algorithm (Li and Durbin, 2010), and peaks were detected in each sample separately using the MACS2 algorithm (Zhang et al., 2008) (using the broad peak option and a *q*-value cutoff of 0.001). A series of QC steps were employed to identify and remove low quality reads (Landt et al., 2012), and samples that did not reach (i) ≥15 × 106 unique reads, (ii) non-redundant fraction ≥ 0.3, (iii) cross-correlation ≥ 0.03, (iv) fraction of reads in peaks ≥ 0.05 and (v) ≥ 6000 peaks were removed. In total, 669 samples passed quality control. Acetylation at the 9th lysine residue of the histone H3 protein (H3K9ac) domains were defined by calculating all genomic regions that were detected as a peak in at least 100 of the 669 samples (15%). Regions within 100 bp from each other were merged and very small regions of less than 100 bp were removed. Read counts were log2 transformed with the addition of 0.5 with accounting the effective library sizes estimated by trimmed mean of *M* values (TMM) scale-normalization using edgeR software (Robinson et al., 2010). Finally, quantified histone acetylation data were quantile normalized. To remove outlier samples based on quantified histone acetylation data, the statistic di was calculated and samples with a di value outside of 1.5x the interquartile range (*n* = 9) were excluded. Then, biological covariates and technical covariates were removed from histone acetylation data via linear regression. Biological covariates include sex, age at death, and three genotyping PCs. Technical covariates include PMI, study index (ROS or MAP), and quality metrics strongly correlated with PC1 (mean fold enrichment, total number of reads, 50% quantile of the mapping quality of all uniquely mapped unique reads, non-redundant fraction and experimental batch for polymerase chain reaction). Histone acetylation data of 413 individuals with other omics measurements are used in the cross-omic analysis.

### Quantitative Trait Locus (QTL) and Epigenomic Features Mapping

Quantitative trait locus mapping for mRNA levels, DNA methylation, and histone acetylation were conducted using FastQTL software (Ongen et al., 2016) with 1,000 random permutations. For QTL mapping for mRNA, SNPs located within 50 kbp of upstream or downstream of transcriptional start site were used for mapping *cis*-QTL. For DNA methylation and histone acetylation peaks, SNPs located within 5 or 50 kbp of upstream or downstream from the center of each peak were used, respectively. The relatively narrow window of genomic regions for QTL analysis is based on the result from published QTL results from GTEx version 7, and HapMap (Banovich et al., 2014), for gene expression, and DNA methylation, respectively. We found the power of QTL detection for gene expression in cortex regions increases as the decrease of QTL window and maximizes at 5 kbp of genomic windows with about 50% increase in the number of QTLs (**Supplementary Figure 1A**). Although a QTL analysis for H3K9ac has not been conducted, the same trend was also observed for QTLs for an alternative histone acetylation for active transcription in three different cells in BLUEPRINT (Chen et al., 2016) (**Supplementary Figure 1B**). Then, we decided to use a relaxed condition of 50 kbp as a genomic window considered for QTL analysis in this study. Prior to QTL mapping, hidden covariates were removed from each omic data. Hidden covariates for each data type were estimated via PEER (Stegle et al., 2010). Consistent with the previous report (Stegle et al., 2010), removing hidden covariates increased the number of genes/epigenomic features associated with SNPs reaching saturation with the removal of 30, 10, and 10 hidden covariates for gene expression, DNA methylation, and histone acetylation (**Supplementary Figure 2**). We set the significance criteria at a false discovery rate (FDR) of 0.05. To identify epigenomic peaks associated with mRNA levels, we calculated correlations between gene expression levels and epigenomic peaks located within 1 Mbp of upstream or downstream of the transcriptional start site for each gene using MatrixEQTL software (Shabalin, 2012). To control the bias of error rate raised from the difference in the number of peaks around transcriptional start site (TSS) for each gene, we also conducted a permutation-based test to identify the gene associated with at least one epigenetic peak using FastQTL software modified to handle continuous values with 1,000 random permutations. We set significance criteria at FDR of 0.05 in both gene level and peak level. To handle outliers conservatively, mRNA levels and quantities of epigenomic peaks were quantile-normalized before the cross-omics mapping. The Storey’s method (Storey and Tibshirani, 2003) was used to calculate a replication rate (π1) with the previously published eQTL result (Ng et al., 2017). To visualize overlap of genes associated with *cis*-regulatory signals, UpSetR software was used (Conway et al., 2017).

**FIGURE 1:**
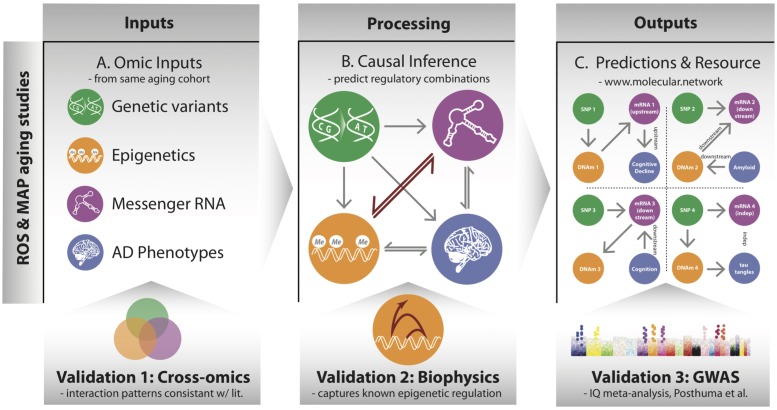
Overview of processing multi-omic data into local regulatory networks (LRNs) and testing the validity of cross–omics interaction. **(A)** Genetic variants and other brain-based omics data were all acquired from the same set of individuals in the ROSMAP aging cohorts. **(B)** Using causal inference methods, we consider many possible networks among the omic data types and infer a likely local regulatory structure around each gene. These networks also include nodes representing AD-related phenotypes. **(C)** The inferred LRNs predict relationships between genes and phenotypes and are publicly available through a web resource: www.molecular.network.

**FIGURE 2:**
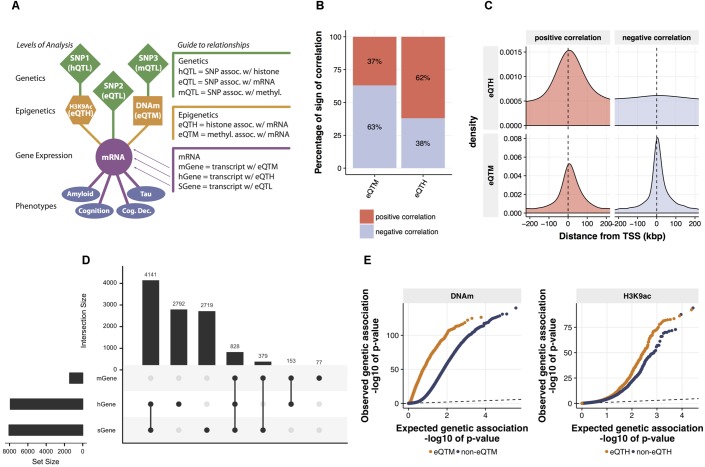
Identification and characterization of relations between mRNA expression and *cis*-regulatory signals. **(A)** The map of cross-omics associations in DLPFC. The *cis*-regulatory associations across mRNA levels, H3K9ac peaks, DNA methylation (DNAm) sites, and SNPs were assessed via linear regression (FDR < 0.05). Brain phenotypes associated with mRNA levels were assessed via Spearman’s correlation (FDR < 0.05). **(B)** Proportions of a sign of the correlation between mRNA levels and both eQTMs and eQTHs. **(C)** Distribution of eQTM and eQTH in relation to transcription start site. **(D)** The intersection of genes associated with cig-regulatory signals. Genes associated with at least one SNP (FDR < 0.05), genes associated with at least one DNA methylation locus (FDR < 0.05), and genes associated with at least one H3K9ac peak (FDR < 0.05) are defined as sGene, mGene, and hGene, respectively. To visualize the number of overlapped genes, UpSet plot was used. The horizontal bar plot represents the total number of sGene, hGene, or mGene. The vertical bar plot represents the number of genes shared among combinations of sGene, hGene, and mGene. For instance, the first column indicates the number of genes shared between hGene and sGene and the forth column indicates the genes shared by all three groups. This “UpSet” plot was generated using UpSetR software (Conway et al., 2017). **(E)** Quantile–quantile (Q-Q) plots of genetic associations of DNAm sites and H3K9ac peaks stratified by their associations to mRNA levels. The significance of genetic associations was assessed via linear regression using FastQTL software with 1,000 times permutation.

### Construction of Local Regulatory Networks (LRNs)

To infer the structure of LRNs, we used a Bayesian network, which is a multivariate probabilistic model whose conditional independence relations can be represented graphically by a directed acyclic graph (DAG) with *vertices* (*V* = *V*_1_,…,*V_p_*), and *directed edges* (*i, j* ∈ *E* ⊂ *V* × *V*) (note that we use the notation *i* and *V_i_*, interchangeably, to refer to a node). A vertex *j* in a DAG *G* corresponds to a random variable *X_j_* in the Bayesian network. Assuming the local directed Markov property, each variable is independent of its non-descendant variables conditional on its parent variables. Thus, the state of *X_j_* can be determined only by the state of parent variables, which is formally expressed by the conditional probability, *P*(*X_j_*|*X_G_j__*) where *X_j_* state occurs under given parents’ state *X_G_j__*. Therefore, the probability where observed data, *X*, is generated from a given DAG *G* can be factored as $P(X|G)=\Pi_{j=1}^{p}P\left(X_{j}|X_{Gj}\right)$ where *X* = (*X*_1_, …, *X_p_*)*^T^*, *G_j_* is the set of parents of *j*, and *X_G_j__* = {*X_i_* : *i* ∈ *G_j_*}. To learn DAG structure, which is essentially the process of finding *G* with high *P(X|G)*, we used a Markov chain Monte Carlo (MCMC) method to sample DAGs based on the posterior distribution of DAG structures

$$P\left(G|X\right)=\frac{P\left(X|G\right)P\left(G\right)}{\Sigma_{G\epsilon g}P\left(X|G\right)P\left(G\right)}$$

where *P*(*G*) is a prior on the network structure *G*, and *g* represents the space of all DAGs with *p* vertices. The MCMC sampling allows us to obtain ensembles of DAGs with high *P*(*X*|*G*) and avoid overfitting to the data. LRNs consist of six types nodes including phenotype (*p*), mRNA levels (∈), DNA methylation levels (*m*), histone acetylation levels (*h*), and SNPs associated with mRNA levels (*g_e_*), DNA methylation levels (*g_m_*), or histone acetylation levels (*g_h_*), and hidden covariates used for the QTL mapping (*C_e_,C_m_,C_h_*). For each variable, hidden covariates were combined as *C_e_j__* = ∑*_i_ w_e_ij__F_ei_, C_m_j__* = ∑*_i_ w_m_ij__F_mi_*, and *C_h_j__* = ∑*_i_ w_h_ij__F_hi_*, where *w_ij_* represents the weight of *j*th variable for *i*th peer factor (*F_i_*). Both *w* and *F* were estimated via PEER method as described in the method of QTL mapping. To utilize SNPs information as a clue to infer the directions of other edges, we restricted a direction of edges so that SNPs can have only out-going edges to other nodes. For non-genetic variables, the parent set used for each node type is as follows;*P*(*e*) ∈{*g_e_, m, h, p, C_e_*}, *P*(*m*) ∈{*g_m_, e, h, p, C_m_*}, *P*(*h*) ∈{*g_h_, m, e, p, C_h_*}, and *P*(*p*) ∈{*g_e_, g_m_, g_h_, m, h, e*}. The levels of non-genetic nodes were quantile-normalized before applying structural learning. We ran 75,000 steps of Markov chain Monte Carlo sampling using the REV algorithm (Grzegorczyk and Husmeier, 2008) and discarded the first 10% of samples as a burn-in. Then, edge frequencies in the sampled networks were counted and generated a consensus network by taking the regulation that presented the most frequently among the three possible states: node1 regulates node2, node2 regulates node1, and node1 is independent of node2. The detailed implementation of learning network structure based on systems genetics data can be found in the previous work (Tasaki et al., 2015).

### Definition of Relations Between Nodes

If there is a path from node1 to node2 in LRN, node1 is classified as upstream of node2 and vice versa. If there is no path between node1 and node2, these two nodes are classified as independent. In the case of calling genes upstream of phenotype, the genes whose mRNA nodes were directly connected to AD phenotypes with outgoing edges were classified as upstream genes. All analyses on network structure were conducted based on the igraph R package.

### Genomic Annotation Enrichment

Gene models were obtained from GENCODE v14. For each transcript, the region from 3 kbp upstream to 3 kbp downstream of TSS was defined as a promoter region, and the region from transcriptional end site (TED) to 3 kbp downstream of TED was defined as a downstream region. The non-promoter region from TSS to TED was defined as a gene-body region. The remaining regions were defined as intergenic regions. Super-enhancer regions for human brains were obtained from dbSUPER (Khan and Zhang, 2016). The uniformly processed ChIP-seq data from 565 of human TFs was downloaded from GTRD (Yevshin et al., 2017). For the enrichment analysis of gene coordinates and super-enhancers, each H3K9ac peak or DNAm site was assigned to a genomic annotation if the center position of H3K9ac peak or DNAm site is overlapped with the annotation. For the enrichment analysis of transcription factor (TF) binding sites, each H3K9ac peak was assigned to a TF if H3K9ac peak is overlapped with its TF binding region. The enrichment of genomic annotation was assessed by Fisher’s exact test. The significance criteria were set as FDR less than 0.05 for all analyses.

### Gene Set Enrichment Analysis

**FIGURE 3:**
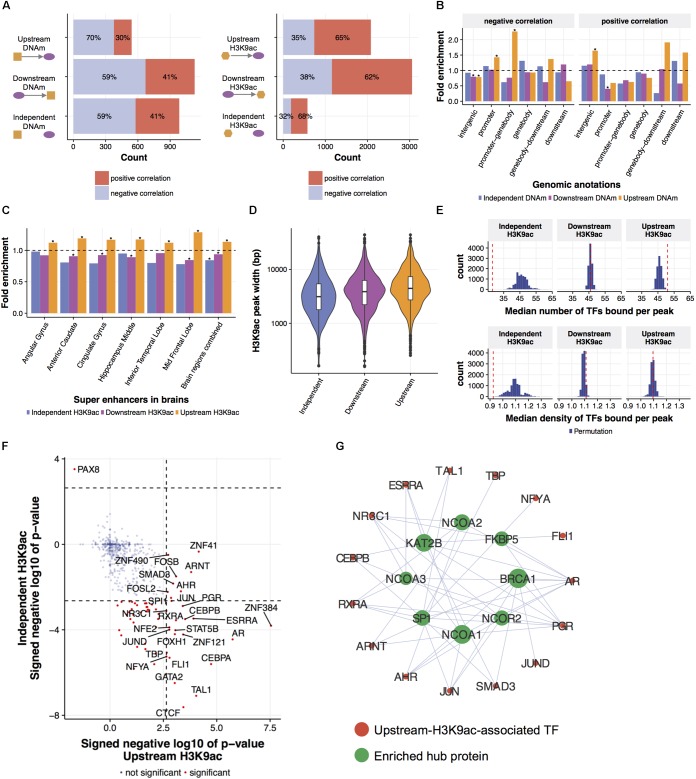
Local regulatory network predicts epigenetic modifications leading gene expression changes. **(A)** Predicted relationships of eQTMs and eQTHs to mRNA levels. An LRN was built for each of 3795 genes and for each AD phenotype, then DNAm and H3K9ac were classified as upstream, independent, or downstream of a given gene expression, based on the direction of the cascade of edges between the mRNA node and both DNAm and H3K9ac nodes. **(B)** The enrichment of predicted relationships of eQTMs to mRNA levels with genomic annotations based on gene coordinate. The asterisks represent the statistical significance based on Fisher’s exact test (FDR < 0.05). **(C)** The enrichment of predicted relationships of eQTHs to mRNA levels with super-enhancers in brains. The asterisks represent the statistical significance based on Fisher’s exact test (FDR < 0.05). **(D)** The distribution of width of eQTHs in relation to their predicted relationships to mRNA levels. **(E)** The TF-binding capability of eQTHs in relation to their predicted relationships to mRNA levels. The upper panel represents the median number of TF peaks overlapping with H3K9ac peaks for each predicted relationship. The lower panel represents the median number of TF peaks overlapping with H3K9ac peaks per 100 bp for each predicted relationship. The histograms represent null distributions estimated by 10,000 permutations of the relations of eQTH nodes to mRNA nodes. The red vertical lines represent observed values based on the estimated directions from eQTHs node to mRNA node. **(F)** The enrichment of TFs binding to eQTHs upstream or independent of mRNA levels. The dashed lines indicate the significance threshold based on Fisher’s exact test (FDR < 0.05). **(G)** TF-TF interaction networks around TFs binding to eQTHs upstream of mRNA nodes. For each TF, the overlap of its binding TFs and the TFs enriched in eQTHs upstream of mRNA nodes was evaluated by Fisher’s exact test (FDR < 0.05).

Gene signatures from public RNA-seq studies were downloaded from Enrichr (Kuleshov et al., 2016). Gene ontology was obtained from the Molecular Signatures Database v6.1 (Subramanian et al., 2005; Liberzon et al., 2011). The multi-validated protein-protein interactions from BIOGRID v3.4.155 (Stark et al., 2006) was used to extract binding proteins for TFs. The enrichment analysis was performed using Fisher’s exact test. Differentially expressed genes (DEGs) for each phenotype were used as the background gene set of the enrichment analysis for upstream genes. For the enrichment of protein interactions with TFs binding to upstream H3K9ac peaks, the 565 TFs in **Figure 3F** were used as a background set. For GO enrichment analysis, all genes in the database were used as a background set. The significance criteria were set as FDR less than 0.05 and the number of overlapped genes greater than 2.

### Genome-Wide Association Study (GWAS) Enrichment Analysis

The summary statistics of GWASs for AD and general cognition were downloaded from http://web.pasteur-lille.fr/en/recherche/u744/igap/igap_download.php, https://ctg.cncr.nl/software/summary_statistics, and https://www.thessgac.org/data. The coloc algorithm (Wallace et al., 2012) was applied to summary statistics of ROSMAP eQTL and GWAS with default parameters of coloc R package. Then, genes showing the strongest posterior probability in the co-localized model were defined as co-localized genes and assessed enrichment of those genes in upstream genes of phenotype by hypergeometric test.

### Data Availability

The datasets analyzed for this study can be found in the Synapse repository (http://dx.doi.org/10.7303/syn3388564, http://dx.doi.org/10.7303/syn3157329, http://dx.doi.org/10.7303/syn3157275, and http://dx.doi.org/10.7303/syn4896408).

## Results

### Summary of Approach

The overarching goal of our approach is to identify the cascade of molecular events that drive age-associated neuropathologies and cognitive decline. To do so, we integrated a large multi-omics dataset from aged brains, which includes genetic variants, DNA 5C methylation (DNAm), acetylation at the 9th lysine residue of the histone H3 protein (H3K9ac), mRNA, and phenotypes via a network-based approach that describes the local regulatory control over the expression of individual genes in aged brains (**Figure 1**). The genomic variants were measured by SNP arrays from blood or brain samples, and the multiple omics data of DNAm, H3K9ac, and mRNA were all assayed in DLPFC from the same set of 413 participants (**Supplementary Table 1**) from the ROS and Rush MAP cohorts, collectively referred to as ROSMAP (see methods). Our analysis consists of three steps. First, this set of omics is unified based on correlation to understand cross-omics relationships among genome, epigenetic marks, and mRNA (**Figure 1A**). Second, those correlative relations are further refined as directed regulatory networks by inferring their conditional independence relations through a Bayesian structure learning framework. We validate directed edges between epigenetic marks and mRNA with existing knowledge on transcription (**Figure 1B**). Third, these networks are further utilized to predict for each gene whether it is “upstream” or “downstream” of disease phenotypes, such as cognitive decline (CogDec) (**Figure 1C**).

### *Cis*-Genomic Features Associated With mRNA Expression Levels

Our model builds a LRN for each gene: these networks capture the impact of genetic and epigenetic variation on the expression of the gene. To ensure these results are biologically plausible, we first surveyed cross-omics correlations among 7,159,943 (imputed with the 1000 Genomes reference) genetic variants, 191,590 DNAm loci, 25,611 H3K9ac peaks, and 12,742 mRNAs. We assessed pairwise correlations between gene expression and *cis*-DNA methylation, or *cis*-H3K9ac that are located, restricting our analysis within 1 Mbp upstream or downstream of each TSS. We identified 1,437 DNA-methylation-associated genes and 7,914 H3K9ac-associated genes with an FDR of 5%. We also used a standard way for mapping quantitative trait loci (QTLs) at multiple molecular levels (xQTL) (Ng et al., 2017) to identify genes, DNAm loci, and H3K9ac peaks associated with *cis-*SNPs. We used a 50Kb *cis-*window to test for eQTLs and H3K9ac peaks, and a 5 Kb *cis-*window for DNAm loci based on results from other studies (see Materials and Methods). We found 8,067 genes, 84,770 DNAm loci, and 7,548 H3K9ac peaks are correlated significantly (FDR < 0.05) with the genotype of their proximal SNPs (**Figure 2A**). This result of QTL mapping is consistent with the associations previously reported based on the same data from ROSMAP participants (Ng et al., 2017) as replication rates (π1) are greater than 0.99 for all three types of omics measurements (**Supplementary Figure 3**), which ensures the quality of normalization and association procedures.

Before estimating LRNs based on the cross-omics correlations, we first conducted a series of characterizations and validations for the observed mRNA-epigenetic correlations in aged brains to ensure correlations reflect the mechanisms involving gene transcription. We examined the direction of mRNA-epigenetic relations and their genomic locations in relation to each TSS. For *cis*-DNAm correlated with mRNA (eQTM) as expected, negative correlations are more common (63%) than positive correlations (binomial test; *p*-value < 2.2e-16) (**Figure 2B**). However, we also observe that DNA methylation at a sizable fraction of eQTMs (37%) is associated with positive gene expression, an observation previously reported (Gutierrez-Arcelus et al., 2013). By contrast, *cis*-H3K9ac correlated with mRNA (eQTH) tends to be positively correlated with mRNA levels (62%) (binomial test; *p*-value < 2.2e-16) (**Figure 2B**), which agrees with findings that H3K9ac is a marker for chromatin undergoing active transcription (Ernst et al., 2011). Indeed, the two marks were chosen in part based on the fact that they are known to be associated with relatively closed and open chromatin states, respectively. The eQTHs associated with positive gene expression are located at the regions close to TSSs, whereas negatively correlated eQTHs are distributed broadly across *cis*-genomic regions (**Figure 2C**). Alternatively, the eQTMs negatively correlated with mRNA levels are more condensed at the TSS regions than the ones associated with positive gene expression (Wilcoxon rank sum test; *p*-value = 3.8e-05) (**Figure 2C**). The enrichment of eQTHs and eQTMs in the key genomic element of transcriptional activity indicates that correlations observed between epigenome and gene expression are likely to be induced by cause-and-effect relationships rather than by lateral confounding factors.

After assessing the independent effects, to quantify the combinatorial regulatory influences on mRNAs, we examined whether mRNA levels are associated with singular SNP, DNAm, and H3K9ac *cis*-signals, or multiple *cis*-signals. Pair-wise significant overlaps are observed between genes regulated by SNPs (sGene) vs. DNA-methylation-associated genes (mGenes) (Fisher’s exact test; *p*-value < 10e-16) and mGenes vs. H3K9ac-associated genes (hGenes) (Fisher’s exact test; *p*-value = 10e-6), but not between sGenes vs. hGenes (Fisher’s exact test; *p*-value > 0.05) (**Figure 2D**). Moreover, genes whose mRNA levels are associated with all three *cis*-genomic features are more frequent than expected by chance (permutation test, *p*-value < 0.0001). This indicates that mRNA levels are more likely to associate with multiple *cis*-genomic features investigated in this study, consistent with our understanding that regulation of mRNA is a coordinated process, rather than conducted by a single source of *cis*-genomic features. As the number of regulatory elements assayed in this cohort increases, we expect further diversification in the origin of regulatory signals.

Having assessed the co-regulatory effects, next we examined whether genetic variations could associate with the relationships between epigenetics and gene expression. Specifically, we contrasted p-values for association with genetic variations between eQTMs and non-eQTMs. We found that eQTMs are more likely to be associated with genetic variants (mQTLs), compared to non-eQTMs (Wilcoxon rank sum test; *p*-value < 10e-16) (**Figure 2E**). We also observed the same trend of genetic influence on eQTHs (Wilcoxon rank sum test; *p*-value = 2.9e-08) (**Figure 2E**). These results suggest that the epigenetic modifications that are associated with genetic variations are more likely to have a functional influence on gene expression in aged brains.

Taken together, our multi-omics data measured in the same set of people reveals reasonable cross-omics relations, which would allow us to learn the characteristics of *cis*-mechanisms regarding mRNA regulations in aged brains via LRNs.

### Assign Directionality of *Cis*-Regulatory Elements via Local Regulatory Networks

Because cross-omic associations are determined based on correlation analysis (**Figure 2A**) it is difficult to determine their causal relationships, except for those with genetics, where we can assume the SNP effect precedes all other effects. Such unidirectional genetic information can be used as a causal prior to predict the relationships between biological measurements (Schadt et al., 2005; Zhang et al., 2013; Tasaki et al., 2015). We developed a Bayesian network (BN) inference method that integrates SNP and omics data (Tasaki et al., 2015) to estimate directed molecular networks. Applying the BN method to multi-omics data setallows us to reconstruct LRN for each gene that models causal relationships between epigenetic modifications, mRNA levels, and phenotypes in aged brains (**Figure 1**). These LRNs consist of nodes for phenotype, mRNA, mRNA-associated epigenetic marks, SNPs associated with levels of mRNA and epigenetic marks, and hidden factors used for QTL mapping (see Materials and Methods). The number of epigenetic marks in LRN varies depending on genes and the best SNP for each variable was included in LRN. To reduce the computational complexity of BN inference, we only included SNP-associated epigenetic modifications in each LRN as we identified those features are more likely to influence gene expression (**Figure 2E**). After this variable selection procedure, 3,795 genes that are associated with both SNPs and one of the epigenetic marks were applied to our BN inference procedure to investigate cross-omics LRNs. Genes used for LRN are not enriched or depleted in any gene ontology categories (FDR > 0.05), suggesting gene selection does not bias biological functions that can be investigated by LRN. Each LRN was estimated with each of the age-related neuropathologies and cognitive phenotypes, PHFtau-tangles, β-amyloid, cognition, and CogDec, resulting in estimating 15,180 LRNs in total.

First, in order to characterize and validate regulations from epigenomes to gene expression, we investigated directed links between mRNA and epigenetic modifications. Based on patterns of connectivity between different types of omics in LRNs, we classified relations between gene expression and epigenetic modifications into “upstream,” “downstream,” or “independent.” Specifically, if there is a path from an epigenetic node to a mRNA node in LRN, an epigenetic node is classified as “upstream.” Conversely, if there is a path from a mRNA node to an epigenetic node in LRN, an epigenetic node is classified as “downstream.” Lastly, an epigenetic node is classified as “independent” if there is no path between an epigenetic node and a mRNA node. Estimated cause-and-effect relations are consistently identified across LRNs with four different phenotype nodes (**Supplementary Figure 4**). Specifically, 2,655 relations between gene expression and DNAm and 5,716 relations between gene expression and H3K9ac are found in at least three out of four LRNs with different phenotype nodes (**Figure 3A** and **Supplementary Table 2**). The consistency of these relationships is higher than expected by chance (permutation *p*-value < 0.0001).

To evaluate the validity of estimated relations of epigenetic modifications to gene expression, we conducted a series of assessments based on biological knowledge that is not included in the process of LRN construction. First, we observed that the excess of DNAm sites that are predicted to be upstream of gene expression are suppressors of gene expression (Fisher’s exact test; *p*-value = 8.3e-06) (**Figure 3A**). Moreover, these suppressive DNAm sites are located in the promoter regions more frequently than DNAm sites that are predicted to be independent or downstream of gene expression (**Figure 3B**). DNAm sites predicted to be activators of gene expression are enriched in distal intergenic regions (**Figure 3B**). The conversion of the effect of DNAm based on the proximity to the promoter is demonstrated by direct editing of DNAm levels by Cas9-fused DNAm modifiers (Liu et al., 2016), and this indicates that LRN models capture known biology regarding the effect of DNAm on gene transcription.

H3K9ac peaks that are predicted as upstream of mRNA nodes in LRNs contain the similar proportion of positive and negative regulators of gene expression compared to other classes of H3K9ac peaks (**Figure 3A**). However, we found that upstream H3K9ac peaks are enriched in the super-enhancers in various brains regions profiled in BI Human Reference Epigenome Mapping Project (Bernstein et al., 2010; Khan and Zhang, 2016), especially in the middle frontal lobe, corresponding to the origin of omics data (**Figure 3C**). Super-enhancers are the cluster of transcriptional enhancers recruiting many TFs and thus have strong transcriptional activities (Hnisz et al., 2013). Since super-enhancers are expected to be larger than normal enhancers (Pott and Lieb, 2015), we asked whether the width of H3K9ac peaks that drive gene expression changes are different from other H3K9ac peaks. As expected, upstream H3K9ac peaks are wider than H3K9ac peaks that are downstream or independent of gene expression (Welch’s *t*-test; *p*-value < 2.2e-16) (**Figure 3D**). To further characterize transcriptional capability of upstream H3K9ac peaks, co-localization of transcriptional factor (TF) binding sites with H3K9ac peaks were investigated by integrating publicly available ChIP-seq data from 565 human TFs (Yevshin et al., 2017). We found that a greater number of TFs are bound to upstream H3K9ac peaks with a median of 51 binding sites (permutation *p*-value = 0.0009), whereas TFs are depleted from independent H3K9ac peaks (permutation *p*-value < 0.0001) (**Figure 3E**). To clarify whether these observations are because of the difference of peak width, we also calculated TF binding density in H3K9ac peaks. TF binding densities in upstream H3K9ac peaks are not significantly higher than others, but those in independent H3K9ac peaks are still significantly lower (permutation *p*-value = 0.0005) (**Figure 3E**). These results indicated that LRN approaches can assign directionality of relations based on the transcriptional capability of H3K9ac peaks in a purely data-driven way without any prior biological knowledge.

We further examined the co-localization of binding sites of individual TF with H3K9ac peaks and identified 28 TFs enriched in upstream H3K9ac peaks and 55 TFs depleted from independent H3K9ac peaks (FDR < 0.05) (**Figure 3F**). We found that these enriched TFs interact with chromatin remodeling machinery in protein levels (FDR < 0.05) (Stark et al., 2006) (**Figure 3G**). One of the hub proteins interacting with enriched TFs is KAT2B (*p*-value = 10e-4), a histone acetyltransferase that mediates acetylation of H3K9: a histone mark integrated into the LRN (**Figure 3G**). This suggests that KAT2B protein induces acetylation of upstream H3K9ac peaks as well as recruits the variety of TFs to regulate gene expression levels in aged brains.

Finally, we examined the similarity and relatedness of LRN-based link predictions with results from correlation-based analysis. DNAm sites that are independent of gene expression in LRNs showed less evidence of correlation with gene expression than upstream and downstream DNAm sites (Wilcoxon rank sum test; *p*-value = 0.0002), but upstream and downstream DNAm sites showed similar levels of significance (**Supplementary Figure 5**). Notably, H3K9ac peaks that are independent of gene expression are more strongly correlated with gene expression levels than upstream and downstream H3K9ac peaks (Wilcoxon rank sum test; *p*-value = 1.0e-12) (**Supplementary Figure 5**), despite their limited activity for regulating gene transcription as suggested above. This indicates that multi-omic integration can distinguish cause-and-effect relations to a greater extent than traditional correlation-based analysis.

### Multi-omic Regulatory Networks Predict Upstream Genes for Age-Related Neuropathologies and Cognitive Phenotypes

**FIGURE 4:**
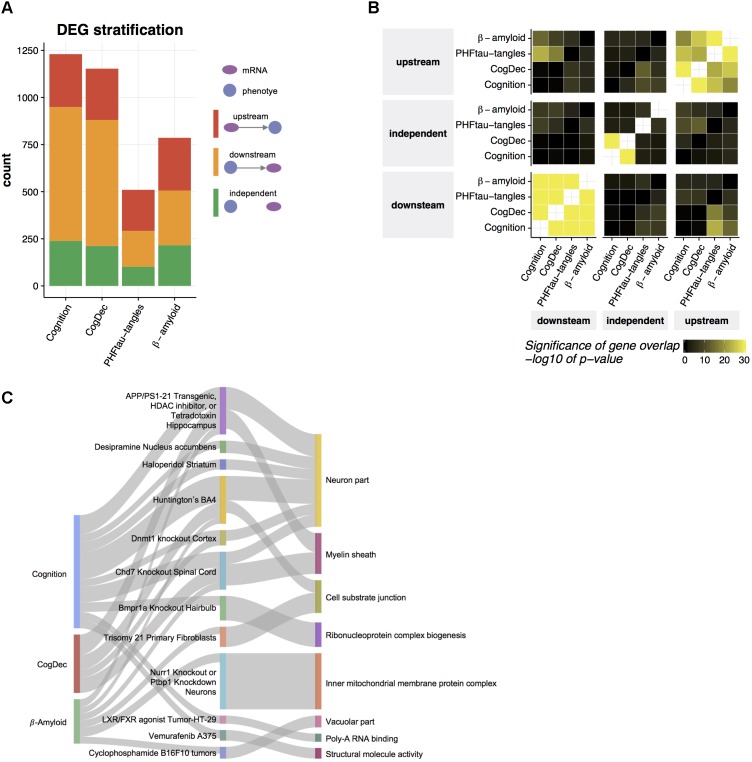
Prediction of upstream genes for neuropathologies and cognitive measures. **(A)** Predicted relationships of DEGs to neuropathologies and cognitive measures. An LRN was built for each DEG and for each neuropathology or cognitive measure, and then genes were classified as upstream, independent, or downstream of a given phenotype, based on the direction of the edge between the mRNA node and the phenotype node. **(B)** Common upstream and downstream genes across phenotypes. Pairwise overlaps among genes predicted as upstream, independent, and downstream of each phenotype were evaluated by hypergeometric test. **(C)** Gene set enrichment for upstream genes. The enrichment of gene signatures from public RNA-seq studies with upstream genes was assessed by hypergeometric test. The top 10 gene signatures significantly associated with the genes upstream of phenotype (FDR < 0.05) were depicted in the middle layer. Gene signatures from the same tissue or cell type were displayed as one category. The thickness of edge corresponds to the fold enrichment. Gene signatures for public RNA-seq studies were further annotated based on their enrichment of gene ontology (GO) terms. If a gene signature was significantly enriched with a given GO term (Bonferroni corrected *p*-value < 0.05) and the overlapped genes contain at least one of upstream genes, an edge between the study name and the most enriched GO term is depicted.

Transcriptome data allows us to understand genes differentially expressed in aged brains with cognitive impairment, which is difficult to achieve based on genetic data because the genome is relatively stable across the lifespan. However, selecting therapeutic targets based on the output of DEG analysis can be challenging because DEGs may indeed be causally upstream of a phenotype of interest (upstream), but in other cases, some or all of those genes may be downstream of the phenotype (downstream). A third possible explanation for observed gene expression changes is that they are in fact independent of the phenotype (independent), but synchronized to it through the action of some third unmeasured latent variable that jointly affects the phenotype and gene expression. Based on the structures of the LRNs for DEG’s of each phenotype, we identified genes which are upstream of cognition, CogDec, β-amyloid and PHFtau-tangles, downstream of the phenotypes, and independent of the phenotypes. Two hundred and eighty-one genes (23% of DEGs), 272 genes (24% of DEGs), 280 genes (36% of DEGs), and 218 genes (42% of DEGs) are estimated as upstream of cognition, CogDec, β-amyloid and PHFtau-tangles, respectively, while the 37% to 58% of remaining DEGs are classified as downstream of phenotypes (**Figure 4A** and **Supplementary Table 3**). The relationships of genes with phenotypes tended to be consistent across different phenotypes (**Figure 4B**), suggesting LRNs robustly identified key genes common for multiple AD-related phenotypes. This is expected given the inter-correlation of the phenotypes.

To understand biological functions related to upstream genes for AD-related phenotypes, we examined overlaps of upstream genes with the collection of gene signatures from 651 RNA-seq studies (Kuleshov et al., 2016) (**Figure 4C**). Thirty-eight gene signatures from 24 RNA-seq studies depicted in the middle layer of **Figure 4C** are enriched with upstream genes for cognition, CogDec, or β-amyloid compared to downstream and independent genes in DEGs for each phenotype (FDR < 0.05, **Supplementary Table 4**). Of these, 13 studies are derived from the brain- or neuron-related studies, such as gene signatures from Huntington brains, hippocampus region of *APP*/*PSEN1* transgenic mouse, and motor neurons with TDP43 knockdown. As expected, these enriched gene signatures are associated with gene ontology categories (Subramanian et al., 2005; Liberzon et al., 2011) related to neuron and myelin systems (**Figure 4C**). This suggests that the upstream genes represent the alterations of neuronal activities.

### Upstream Genes Are Enriched in GWAS of Human General Cognition

**FIGURE 5:**
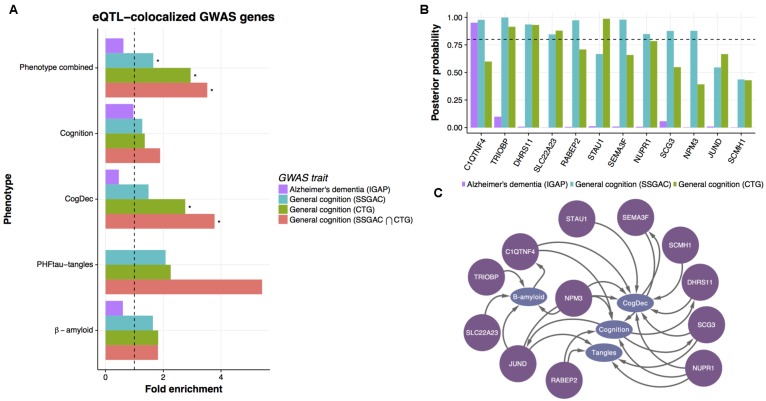
Enrichment of general cognition GWAS signals in upstream genes. **(A)** The enrichment of genetic associations in upstream genes based on eQTL co-localized genes. The enrichment of genes showing co-localized eQTL and AD or general cognition GWASs in upstream genes was evaluated by hypergeometric test. An asterisk indicates *p*-value < 0.05. **(B)** The posterior probability of co-localization for genes enriched in upstream genes. **(C)** Predicted relationships between genes colocalized with general cognition GWASs and phenotypes in LRNs.

To evaluate the prediction of upstream genes, we assessed whether the GWAS genes associated with cognition or AD are concentrated in genes predicted to be upstream of phenotypes. For this assessment, we used genetic associations with clinical AD diagnosis from the International Genomics of Alzheimer’s Project (IGAP) (Lambert et al., 2013) and those from two meta-analyses of GWAS for human general cognition (Sniekers et al., 2017; Lee et al., 2018). Although two general cognition GWASs potentially share part of participants through the UK Biobank, we used these two recent GWASs to increase the robustness and generality of results. The biological implications based on primary genetic findings from these studies are different: AD GWAS shows the contribution of immune-related genes to clinical AD diagnosis whereas general cognition GWASs indicates the critical roles of neuronal genes in cognitive performance. These panels allow us to evaluate the upstream genes from distinct biological perspectives. To compare upstream genes with GWAS results, we assessed GWAS signals in upstream genes based on a detailed spatial association between eSNPs for upstream genes and GWAS signals in those genes. Specifically, we assessed co-localization of eQTL signals from ROSMAP data and GWAS signals of AD and general cognition by the “coloc” algorithm (Wallace et al., 2012). The coloc algorithm estimates posterior probabilities for the model where eQTL signals are co-localized with GWAS signal and the models where these signals are not co-localized. Within the DEGs for any of four phenotypes, eQTL signals of 9, 24, and 74 genes are co-localized with AD GWAS, and two general cognition GWASs, respectively (**Supplementary Table 5**), and those genes are likely controlled by causal SNPs in DLPFC. Interestingly, the co-localized gene sets from two cognition GWAS are both significantly enriched with the upstream genes for any of four phenotypes compared to downstream or independent genes [**Figure 5A**; hypergeometric test; *p*-value = 0.009 and 0.02 for Sniekers et al. (2017) and Lee et al. (2018), respectively], but the gene set from AD GWAS is not (*p*-value = 0.85). As expected, the co-localized genes from two general cognition GWASs are overlapped significantly (**Supplementary Figure 6**). Then, we further examined the enrichment of upstream genes with 17 genes that are identified in both studies and observed an increase in fold enrichment (**Figure 5A**). We then broke down these associations into each phenotype and found that the upstream genes for each phenotype tend to enrich with the colocalized genes for general cognition, in particular for CogDec (**Figure 5A**). The result supports causal roles of upstream genes for cognitive processes. The smaller overlaps of AD GWAS and predicted upstream genes in DEGs is also suggested by a previous analysis of the ROSMAP transcriptome that found the immune gene signature enriched with AD GWAS was associated with age, but not AD-phenotypes (Mostafavi et al., 2018). Conversely, as both upstream genes and the primary findings from general cognition GWASs are characterized by the involvement of neuronal genes (**Figure 4C**), two complementary approaches point to the coherent cellular component regarding cognitive phenotypes.

Of the consensus colocalized genes in general cognition GWASs that are overlapped with upstream genes, ten genes show strong evidence of co-localization (posterior probability > 0.8) (**Figure 5B**) in either study. Those genes are mostly predicted as upstream of cognition or CogDec (**Figure 5C**), suggesting that they are top candidates of genes affecting cognitive performance possibly accompanied by the pathological burden. Particularly, literature evidence suggests that *STAU1* and *SEMA3F* play critical roles in synaptic transmission and neural circuits formation (Sahay et al., 2005; Lebeau et al., 2008). These results, showing overlap with causal variants defined by large general cognition GWASs, indicate that the multi-omic network framework is likely to provide a novel approach to prioritize DEGs for further validation experiments.

## Discussion

Overall, this method of predicting multi-omic networks provides a detailed description of gene regulation across the genome in aged brains and capitalizes on the original promise of omics to improve our understanding of disease. Importantly, these molecular regulations were inferred based on data from DLPFC in older adults, thus this ensures these results describe molecular events existing in a brain region relevant to cognition and cognitive decline. The mathematical method by which we do this reaches back to the genome for causal anchors and then flows forward through epigenomes, gene expression to pathological and clinical AD phenotypes. These predictions of cross-omics interactions are likely to be accurate, not only because they incorporate diverse sources of information, but also because they are concurrent with biological knowledge on epigenetics and causal information from a related phenotype. Specifically, multiple rounds of validation, from the variety of genome annotations, large-scale ChIP-seq compendia, general cognition GWASs, and co-localization, all indicate the predicted multi-omic networks accurately capture some aspects of biological regulation.

Determining the downstream effects of epigenetic changes on gene expression levels are one of the challenges in the study of the epigenome. Despite its importance, computational approaches to address this question have not been well studied yet (Gutierrez-Arcelus et al., 2013). Our multi-omic integration predicts epigenetic peaks driving gene expression and we successfully show their prominent transcriptional capability based on the location of peaks and TF binding capabilities (**Figure 3E**). These results are the most extensive attempt to infer the consequence of alterations of DNAm and H3K9ac. These predictions can be supplied to recently developed Cas9 systems that can modify epigenomes for further validation (Liu et al., 2016; Kwon et al., 2017). The results from experimental validation can be used as causal priors for reconstructing directed networks, which allows us to improve the accuracy of our LRN estimation iteratively. Our catalog of multi-omics LRNs provides the first hypothesis landscape of causal epigenome-transcriptome associations and will help to boost functional understanding of epigenomes in humans.

These upstream genes are enriched with neuronal signatures driven by genetic or compound interventions (**Figure 4C**). Of these interventions, knockout mutations of *DNMT1* cause demented phenotype in humans (Klein et al., 2011), inhibition of *class I HDACs* regulates memory extinction (Gräff et al., 2014), and treatment with tetrodotoxin impairs special memory (Wesierska et al., 2005). Thus, our analysis is likely to capture the genes affecting cognitive performance in broad situations, which concur with the nature of the prospective design of ROSMAP cohort that includes a range of mechanisms affecting cognitive performance (Bennett et al., 2018). Among the upstream genes, in particular, *STAU1* showed strong evidence of genetic association with general cognition (**Figure 5B**). This protein is an RNA-binding protein playing roles in transporting RNA granules along dendrite in neurons and maintaining efficient synaptic transmission in hippocampal synapses (Lebeau et al., 2008). In addition, STAU1 forms a protein complex with TDP43, whose mutation is associated with frontotemporal lobar degeneration and amyotrophic lateral sclerosis and this complex regulates the sensitivity of neuronal cells to apoptosis and DNA damage (Yu et al., 2012). *TRIOBP* is another upstream gene supported by genetics (**Figure 5B**). TRIOBP is a binding protein for TRIO, a guanine nucleotide exchange factor (Seipel et al., 2001) and its mutations are associated with hearing impairment (Shahin et al., 2006). Interestingly, the dysfunction of *TRIO* causes mild intellectual disability (Ba et al., 2016) and Rho GTPases regulated by TRIO are involved in the processes of synaptic loss and β-amyloid production (Schmidt and Debant, 2014). Another interesting gene *SEMA3F* (**Figure 5B**), a secreted member of the semaphorin III family, plays important roles in synaptic transmission and neural circuits formation (Sahay et al., 2005). *SEMA3F* regulates dendritic spine dynamics and hippocampal excitatory networks application to cultured neurons and acute hippocampal slices, respectively (Sahay et al., 2005; Demyanenko et al., 2014). Interestingly, *SEMA3A*, a close member of *SEMA3F* in a semaphorin III family, is associated with neuropathologies in the hippocampus of AD patients (Good et al., 2004). These findings support that the validity of our integrated computational approach to screen genes that influence neuropathologies and cognitive processes based on the posterior probability of an outgoing edge from a mRNA node to a phenotype node in the LRN.

The question often arises about the ability of causal inference methods to recapitulate GWAS hits. We utilized a separate set of subjects and show enrichment of GWAS hits for general cognition, among genes predicted to be upstream of cognition. Two general cognition GWASs do not specifically focus on older adults, but some fractions of participants are likely from older adults with preclinical AD. This might explain the enrichment of general cognition GWAS signals in the genes upstream of cognitive function, as well as common neuronal pathways shared by various conditions with cognitive dysfunctions. The lack of predictions that AD GWAS hits are upstream of cognition should be viewed in the context of the effect of genetics on gene expression in DLPFC. The expression levels of genes located in the vicinity of the robustly validated AD variants are not associated with cognitive decline or AD pathology in DLPFC, but with age (Mostafavi et al., 2018). Because of this limited influence of the known genetic architecture of AD on cognitive decline overall and through gene expression, the absence of AD GWAS enrichment in upstream genes is not surprising, as our prediction of upstream genes assumed significant correlations between genes and the phenotypes. We should note, however, that we successfully identified genes affecting β-amyloid production based on ROSMAP transcriptomes without using genetic information (Mostafavi et al., 2018), indicating genes playing critical roles in AD-phenotypes are not necessarily implicated by genetics and cannot be discovered even by the recent meta-GWAS (Lambert et al., 2013). One of those validated genes, *INPPL1*, has an eQTL and associated epigenetic modifications and thus was investigated with the LRN. Consistent with the result from previous experimental validation, the LRN predicted *INPPL1* as upstream of β-amyloid^1^, which further supports the accuracy of our prediction.

Our analysis focused on LRNs that model multi-layer regulatory networks for a single gene. Although our LRN model captured known biology regarding relationships between, epigenomes, gene expression, and phenotypes, many indirect relations should be included in the single gene LRN because we omit the influence from other genes. Thus, in theory, extending LRN to multi-gene multi-omic networks would improve the accuracy of predictions, however, this requires further development of efficient methods to search a huge possible number of network structures comprising thousands of nodes (Tasaki et al., 2015). Also, the accuracy of regulatory networks and hence of predicted upstream genes should improve with the addition of other omic data, obtained in these same individuals. For instance, integrating microRNA levels, DNA methylation at 5-hydroxymethylcytosine, or other histone marks should lead to more accurate structure in the LRNs, as would information on the activation state of promoters, obtained via ATAC-seq. The inclusion of data from non-Caucasian genetic backgrounds with varying minor allele frequencies could also provide improved predictions. While we provide extensive validation of the causal classification, experimental tests of several predicted upstream genes with novel relevance to cognitive decline will further test the validity of these predictions, and potentially define the drivers of disease mechanisms.

## Author Contributions

ST and CG worked on the study design and manuscript drafting. PDJ and DB acquired the data. ST carried out the data analysis. ST, CG, SM, LY, YW, PDJ, and DB interpreted the data. All authors have critically reviewed the manuscript and approved the final manuscript.

## Conflict of Interest Statement

The authors declare that the research was conducted in the absence of any commercial or financial relationships that could be construed as a potential conflict of interest.

## Footnotes

1 www.molecular.network
