# Supplementary material for: Multi-omic Directed Networks Describe Features of Gene Regulation in Aged Brains and Expand the Set of Genes Driving Cognitive Decline
Source: Front Genet. 2018 Aug 9;9:294. doi: 10.3389/fgene.2018.00294 (PMC6095043; doi:10.3389/fgene.2018.00294)
Supplement: Supplementary file 5 [file Presentation_1.pdf]

n	413
age_death (mean (sd))	88.79 (6.64)
female (n, %)	267 (64.6)
years of education (mean, sd)	16.48 (3.40)
MMSE, last visit (mean, sd)	21.54 (8.89)
Clinical dementia (n, %)	171 (41.4)
MCI (n, %)	111 (26.9)
NCI (n, %)	131 (31.7)
AD (n, %)	160 (38.7)
Global AD pathology (mean, sd)	0.65 (0.58)
Amyloid score (mean, sd)	3.52 (3.75)
Tangles score (mean, sd)	6.18 (7.83)
Presence of gross infarctions (n, %)	145 (35.1)
Presence of microinfarcts (n, %)	107 (25.9)
Presence of Lewy bodies (n, %)	80 (19.4)

Supplementary Table 1. Clinical characteristics of ROSMAP multi-omics cohort. The raw data are available to download through www.radc.rush.edu.

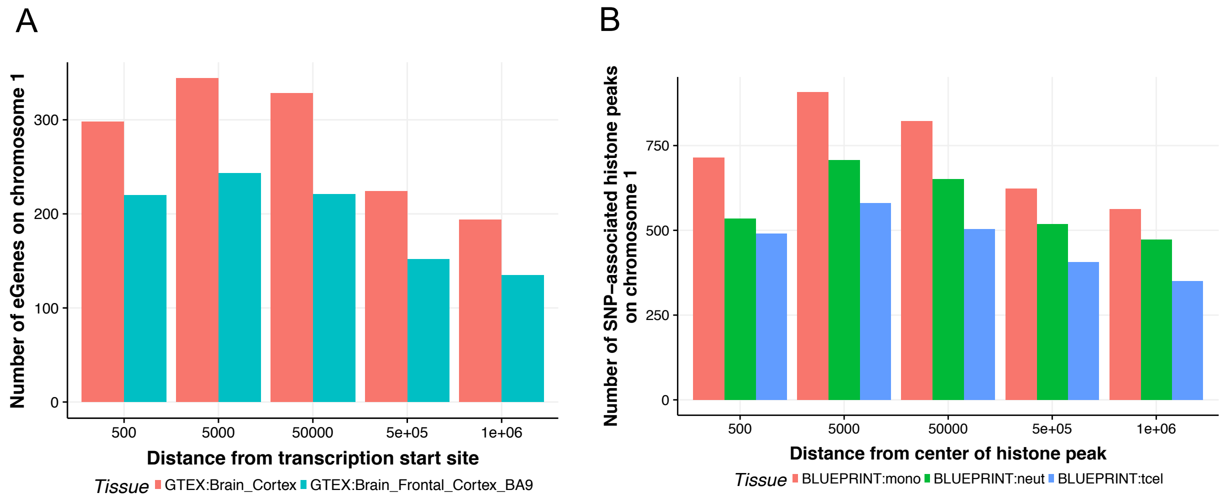

Supplementary Figure 1: The effect of window size on the power for detecting SNP-associated variables.

For each variable, p-values of SNPs in a given widow from TSS or center of H3K27ac peak are subjected to Bonferroni correction and then the SNP showing the minimum p-value is used as a variable-level QTL significance. This variable-level QTL significance was used to calculate FDR for detecting SNP-associated variables. We set the significance criteria at FDR of 5%.

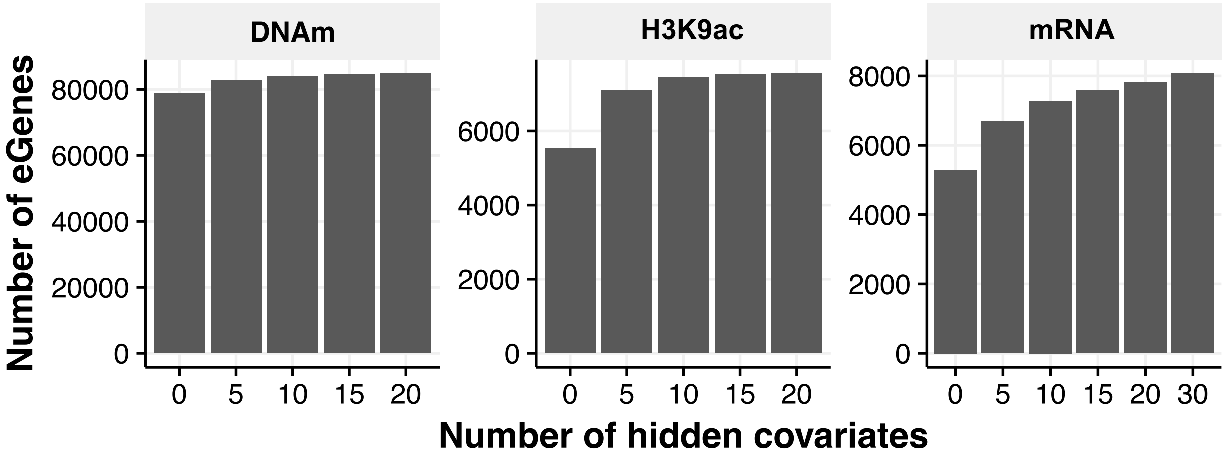

Supplementary Figure 2: The effect of surrogate variables on the power for detecting SNP-associated variables.

The effect of surrogate variables on the power for detecting SNP-associated variables. Gene expression, histone acetylation, and DNA methylation associated with SNPs were evaluated using the fastQTL program with 1,000 permutations (FDR<0.05). The number of surrogate variables removed from each dataset varied from zero to 20 and 30 for epigenome data and gene expression data, respectively.

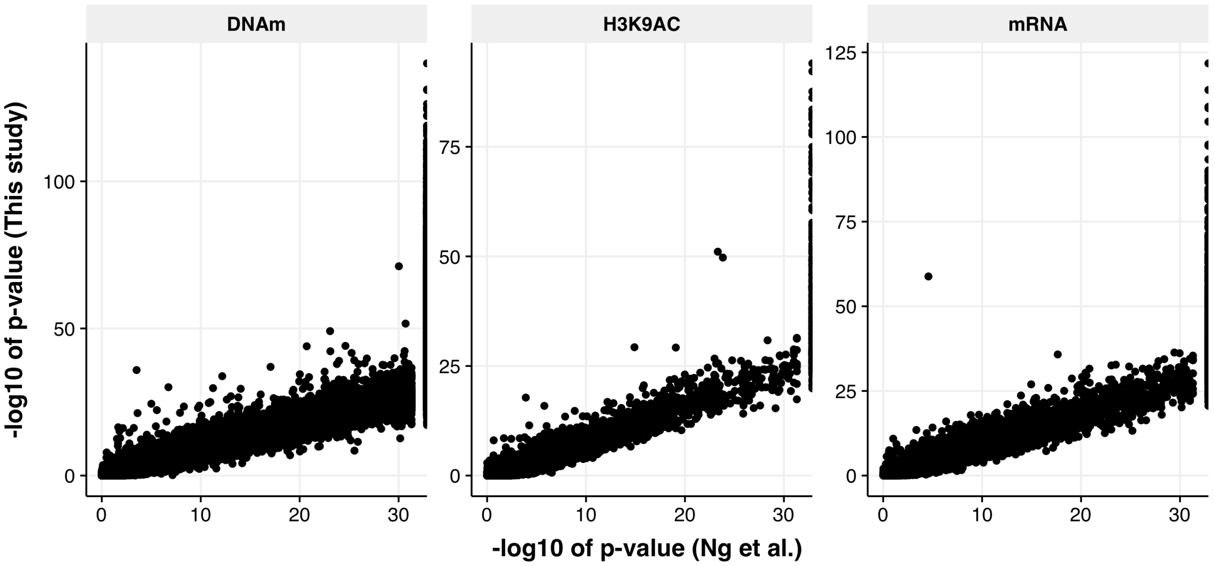

Supplementary Figure 3: Comparisons of xQTL result.

Scatter plots indicate that comparisons of genetic associations of mRNA, DNAm, or H3K9AC between the result from our permutation-based procedure and previous result from Ng et al. based on Spearman's correlation.

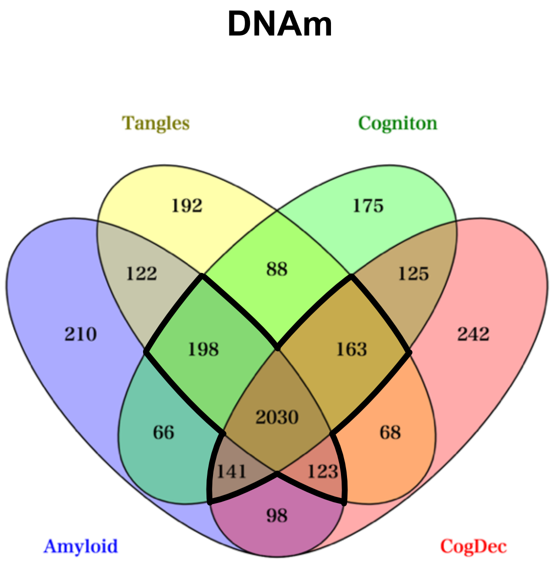

Supplementary Figure 4: The number of consistent relations between epigenomes and gene expression in LRNs with four different phenotypes.

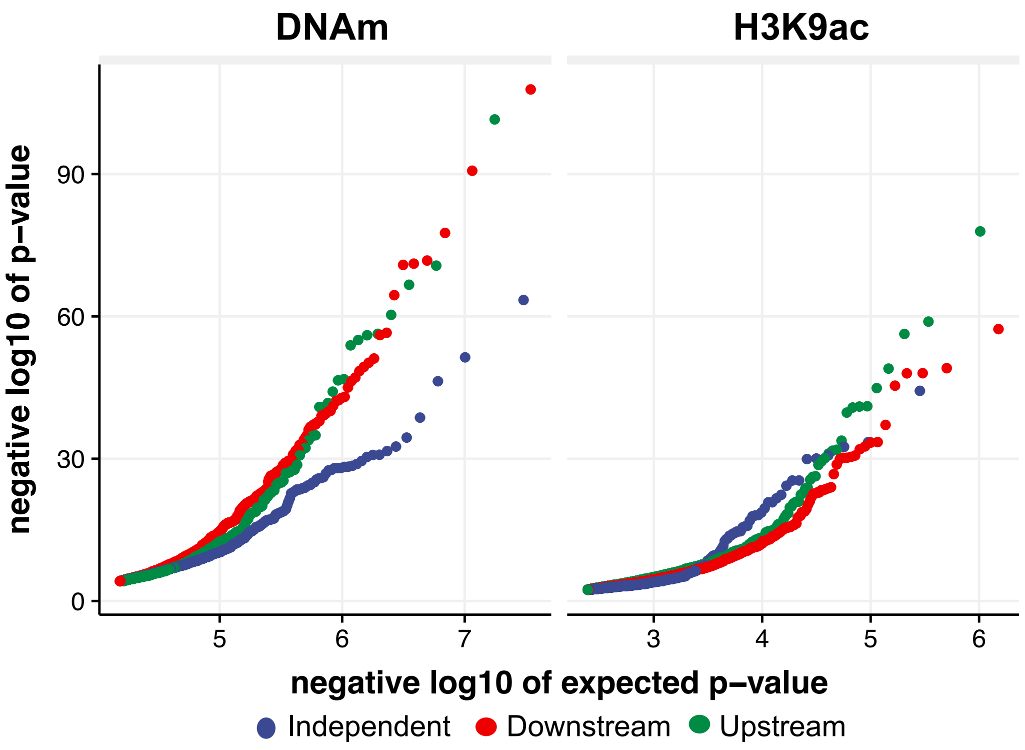

Supplementary Figure 5: Q-Q plot for correlational associations of epigenomes to gene expression. Q-Q plot indicates p-values for QTM and eQTH stratified with predicted relations against mRNA nodes in LRNs.

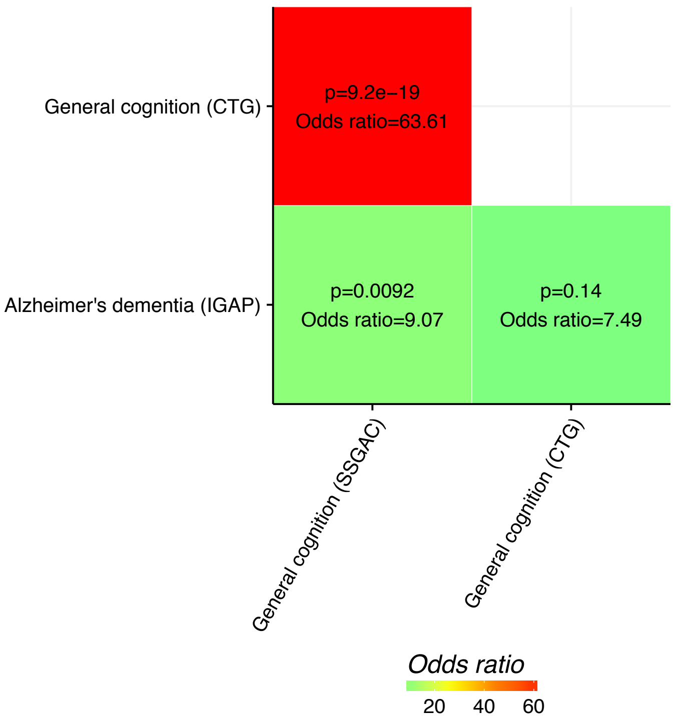

Supplementary Figure 6: Comparison of eQTL-colocalized GWAS genes.

The co-localization of eQTL signals in ROSMAP and GWAS signals was evaluated by the coloc algorithm and the overlap of co-localized genes was evaluated by hypergeometric test.
